# Non-adaptive cognitive emotion regulation mediates the relationship between disease uncertainty and acute stress disorder in patients with ischaemic stroke

**DOI:** 10.3389/fpsyt.2024.1319848

**Published:** 2024-03-05

**Authors:** Yanan Zhou, Yani Zhou

**Affiliations:** ^1^ School of Nursing, Qingdao Binhai University, Qingdao, Shandong, China; ^2^ The department of breast and thyroid, Zhongshan Hospital Affiliated to Dalian University, Dalian, Liaoning, China

**Keywords:** cognitive emotional regulation, ischaemic stroke, disease uncertainty, acute stress disorder, mediating effect

## Abstract

**Background:**

During epidemic outbreaks, hospitalized patients, especially those with cerebrovascular disease, were identified as a vulnerable group suffering from acute stress disorder (ASD) and consequent psychological distress. For stroke patients, not only will they suffer from physical illness, but the uncertainty of illness caused by sudden illness may also cause patients to experience different degrees of ASD. Relevant studies have shown that the impact of ASD on individuals may vary according to age, gender, disease characteristics, individual personality, treatment methods, income level, family support, cognitive psychology and other factors. However, non-adaptive cognitive emotion regulation plays a crucial role in influencing individual psychological states. At present, the risk factors of ASD after stroke and the mechanism between illness uncertainty and cognitive emotion regulation are not fully understood. Therefore, we focus on exploring the predictive effects of general demographic and disease-related characteristics, maladaptive cognitive emotion regulation, and illness uncertainty on ASD after stroke, and make hypotheses. When a disease acts on the body, the patient will have the corresponding cognition of the disease, and ASD will appear at the same time. Then the maladaptive cognitive emotion regulation as an important mediating variable can aggravate the level of acute stress disorder and be verified.

**Methods:**

We used a cross-sectional design, which can be used to investigate the distribution of a disease or health condition and its related factors in a specific population at a specific time, so as to describe the distribution of the disease or health condition and its relationship with related factors. A total of 256 hospitalized patients with ischemic stroke were enrolled, including 145 males and 111 females, aged from 26 to 90 years, with a mean age of (64.71 ± 12.20) years. All patients completed and returned a self-report questionnaire that included demographic information, illness uncertainty, cognitive emotion regulation, and ASD. We then compared the differences in general demographic data, illness uncertainty, and maladaptive cognitive emotion regulation in acute stress disorders.

**Results:**

The majority of hospitalized stroke patients (67.6%) developed ASD due to the COVID-19 pandemic and were therefore at risk for PTSD. More than one third (39.1%) of stroke survivors also suffered from severe psychological distress. More specifically, younger stroke patients are more likely to experience ASD than older patients. Although higher illness uncertainty scores indicate more severe ASD, adaptive cognitive emotion regulation was a protective factor.

**Conclusion:**

Given that individuals with ASD are susceptible to PTSD, it is critical to follow up hospitalized patients with ischemic stroke for screening for PTSD and referral to appropriate psychological services. Maladaptive cognitive emotion regulation can increase the impact of uncertainty on the traumatic experience of stroke patients. Therefore, health care institutions should increase their efforts to provide psychosocial support services to hospitalized patients and make continuous efforts to screen for symptoms of trauma and psychological distress in hospitalized stroke patients.

## 1 Introduction

The speed and aggressiveness of the epidemic virus threaten the lives and property of millions of people around the world, and may cause varying levels of emotional trauma disorder around the world. Due to various reasons, such as worrying about the outcome of the disease, the negative impact of the disease on relatives, and the economic burden beyond the range of their own. Especially for patients with chronic nervous system diseases, the treatment cycle is long, the disease develops rapidly, the treatment methods are complex, and the mortality rate is high, which will increase people’s sense of helplessness and uncertainty. The acute onset of ischaemic stroke can lead to neurological deficits and limited self-care, requiring a long period of treatment and rehabilitation. In the context of the current serious epidemic situation, patients are prone to high levels of acute stress when stimulated by multiple stressors such as sudden unknowns, uncontrollability and uncertainty, which, if not recognized and intervened in a timely manner, can lead to long-term chronic damage and seriously affect the quality of survival (1). Uncertainty about illness is an important psychological stressor that can exacerbate the level of acute stress response, leading to prolonged treatment cycles and long-term health effects (2). Studies have found that stress symptoms are more pronounced in patients with acute illness who have higher levels of uncertainty about their illness (3).

Illness uncertainty is an important part of the patient’s illness experience, which can significantly affect the patient’s psychological adjustment and adaptation ability and even the outcome of the disease. Increasing patient stress in the course of illness (4). Patients are unable to determine the nature of stroke for a long time, can not clear the direction of diagnosis and treatment, and even have no understanding of stroke disease, which leads to long-term anxiety. Patients lack of correct understanding of the disease and the high uncertainty of the disease, which will produce the sense of disease uncertainty, and also reduce the psychological tolerance of patients. As a result, the hope level and coping and adjustment ability of patients decrease correspondingly, and finally aggravates the degree of ASD of patients. Uncertainty in illness can be affected by education level, disease cognition, social and family support and other factors. Although the theoretical research on uncertainty in illness is becoming more and more mature, most of the current research is mainly focused on the research of patients with chronic diseases and cancer, and there are relatively few studies on patients with acute diseases. Patients with many diseases have shown different degrees of illness uncertainty and negative psychological emotions in the early stage of the disease, but there are still few studies in this field for stroke patients.

As an important part of emotion regulation, cognitive emotion regulation plays an important mediating role in multiple negative events and clinical outcomes (5). It has been shown that non-adaptive cognitive regulation can lead to higher levels of stress and affect recovery progress and prognosis (6). Self-regulation theory points out that the patient’s psychological stress state can be corresponding to the mapping of the interaction between the individual, the behavior and the environment. It explains that the individual can output the corresponding adaptive behavior through cognitive or emotional illness perception when facing the stimulation of the internal and external environment. Including individual emotional reactions can affect patients to make different coping behaviors, and finally to evaluate the impact of individual coping behaviors on the disease. If the patient’s psychological stress is not adjusted properly, it will pose a certain threat to the patient’s life and health (7).

In 2001, Garnefski formally put forward the concept of cognitive emotion regulation, which refers to the cognitive effort made by individuals to deal with internal or external life events that are beyond their ability to bear, and the management of emotions from the perspective of cognition, which is easily affected by consciousness, cognitive evaluation and other factors. In order to assess which cognitive emotion regulation mode individuals tend to adopt after experiencing a negative life event, scholars have developed cognitive emotion regulation questionnaire, including adaptive and non-adaptive regulation. At present, the cognitive emotion regulation questionnaire developed by Garnefski is mainly used to evaluate the cognitive emotion regulation ability of patients in the world (8).The nine modules in the questionnaire represent nine different ways of cognitive emotion regulation. Among them, acceptance, positive adjustment, focus on planning, positive reappraisal, and self-comfort belong to the PCER, and self-blame, catastrophization, blame others, and rumination belong to the NCER. It indicates that individuals are more inclined to use the emotion regulation mode represented by this module to cope with stress. Most researchers accept this classification method and verify the influence of various coping styles on individuals. NCER will have adverse effects on individuals. Adaptive emotional expression often wins good social relations, while maladaptive emotional expression often makes people feel psychological pain (9). Stroke patients will have different degrees of uncertainty after illness and face physical and psychological stress. Therefore, it is necessary to clarify the relationship between maladaptive cognitive emotion regulation and ASD, and intervene in time for poor cognitive emotion to alleviate the stress level and promote the prognosis of patients.

In addition, ASD is also influenced by the general demographic and disease status of the individual: related studies of psychological stress have shown that acute stress is significantly higher in female patients than in males (10). Compared with men, women are more likely to use poor psychological adjustment strategies and receive less social support than men, which leads to their higher ASD (11).The female group is not satisfied with the family support and does not get enough emotional value, which will also increase the level of ASD.

The data show that compared with young people, older people are more likely to adopt adaptive psychological adjustment strategies to cope with stress, while young people have poor psychological adjustment ability, which will lead to higher levels of acute stress (12).

At present, income has been widely confirmed as a potential variable affecting psychological status. It has been suggested that higher income has a protective effect against ASD (13). Internally perceived low income significantly predicted ASD. Working status as the first source of income and residence as a social factor to measure the level of income also affect the level of ASD.

At present, the research on the influencing factors of social support for ASD has been relatively mature, and it has been found that it has a strong predictive effect. Ozer et al. tested some adult patients with stress disorder and found that social support could alleviate the specific symptoms of ASD, and the effect was significant, and the effect was cumulative over time (14).

For patients with cerebrovascular disease, the cause and mechanism of the disease directly lead to the consequences of the severity of trauma. The location of ischemia, the degree of nerve defect, the degree of limb numbness, and the number of operations may cause further psychological problems in patients. The disease itself, as a stressor, also always affects the outcome and prognosis of patients. Literature has shown that the susceptibility factors of psychological stress disorder in hospitalized patients are related to personality, and the severity of trauma and pain are the main factors affecting the level of psychological damage of patients. Different patients will have different ASD symptoms due to the nature of stressors and the severity of trauma (15).

We found that, from the perspective of research objects, stroke patients with dysfunction lead to the lack of self-care ability, self-image disorder, self-value can not be realized, etc., leading to patients may suffer from external prejudice and discrimination, so that patients are under high psychological and physical pressure. Therefore, stroke patients with ASD deserve attention. From the perspective of related factors, the influencing factors of stroke ASD are mostly objective factors. Existing studies have found that demographic variables, such as: Age, gender, ethnicity, marital status, personality, education level, economic ability, work status, and place of residence have certain effects on ASD symptoms, as well as disease-related factors such as disease severity. So for stroke patients, we want to hypothesis and test, Whether the objective factors such as gender, age, spouse, introversion or extroversion, income level, treatment cost reimbursement, living in a big city, disease severity, self-care ability, and surgery have predictive effects on ASD in stroke patients, and if so, what is the predictive effect? These were clarified and verified in this study. In addition, ASD is associated with uncertainty, and patients’ sense of certainty and control are damaged after a stressful event. In the existing studies on emotional disorders, quality of life and uncertainty in illness of patients with cerebrovascular disease, anxiety, depression, coping style, social support and so on are related to uncertainty in illness. ASD is also related to coping style and social support. Therefore, it is hypothesized that the occurrence of ASD in stroke patients is related to illness uncertainty and cognitive emotion regulation, which are predictors of ASD level. At present, although the research on uncertainty in illness, cognitive emotion regulation and ASD has made some progress, it is only a single aspect of research. There are few studies on the relationship among uncertainty in illness, cognitive emotion regulation (non-adaptive) and ASD in stroke patients at home and abroad, and the comprehensive relationship among the three and the internal mechanism are not clear. Therefore, it is necessary to explore the relationship among them. Objective factors are often difficult to improve through their own efforts, but subjective factors can be adjusted and intervened by human beings. Therefore, we should pay attention to the patient’s subjective feelings to regulate the patient’s ASD, hoping to improve the patient’s ASD by adjusting the cognitive perspective. Therefore, this paper uses a cross-sectional study to clarify the predictive factors of ASD after stroke, explore the mechanism of action among the three and the true feelings of stroke patients, so as to provide data support and theoretical support for formulating timely and effective intervention support strategies and reducing the level of ASD in patients, so as to promote the rehabilitation of stroke patients. To reduce the recurrence rate of the disease and improve the quality of life of patients.

## 2 Methods

### 2.1 Design

From July 2022 to May 2023, we selected 256 patients with ischemic stroke who were hospitalized in the Department of Neurology of a Classiii Grade A hospital in Jilin Province, China and met the inclusion and exclusion criteria as the research objects by the convenient sampling method. The inclusion criteria were as follows: (1) meeting the diagnostic criteria of Chinese guidelines for diagnosis and treatment of acute ischemic stroke; (2) the first onset; (3) Be aged 18 years or above; (4) 2-28 days after onset; (5) clear consciousness and normal language communication ability. Exclusion criteria were as follows: (1) disturbance of consciousness; (2) impaired cognitive function; (3) craniocerebral trauma, organic brain injury, serious organic lesions of heart, liver and kidney; (4) other severe stressful events occurred recently; (5) schizophrenia, organic psychosis, anxiety disorders and other mental disorders.

### 2.2 Sample and sample size

The inclusion and exclusion criteria were strictly implemented, and the sample size was calculated by the rough estimation method. There were 30 variables in the four survey scales used in this study, and 7 times the number of variables were used in this study. However, considering the missing or invalid questionnaires, the original sample size was increased by 20%. In the actual survey, a total of 260 questionnaires were distributed, 256 were valid and 4 were invalid, so the sample size of this survey was determined to be 256.

### 2.3 Measurements

#### 2.3.1 Sociodemographic data sheet

A total of 11 socio-demographic questions were independently formulated, including gender, age, education level, etc. We also obtained the treatment methods and NIHSS scores of the patients through the doctor’s working system to evaluate the degree of neurological deficit of the patients, and the BI scores of the patients through the nursing records of the nurses to judge the self-care ability of the patients.

#### 2.3.2 Uncertainty in illness

Mishel Uncertainty in Illness Scale-Adult (MUIS-A) was translated by Sheila Sheu, with 25 items. The Likert 5-point scale was used, including 2 dimensions of ambiguity and complexity, with a total score of 25 to 125, divided into 3 levels (16).

#### 2.3.3 Cognitive emotional regulation

Cognitive Emotional Regulation Questionnaire (CERQ) was translated and included 9 dimensions of self-blame, acceptance, contemplation, positive refocusing, refocusing plan, positive re-evaluation, rational analysis, catastrophizing, and blaming others, with a total of 36 items, each dimension containing four items respectively. Each dimension contains four items and is rated on a 5-point Likert scale from “never” to always”, with scores ranging from 4 to 20 for each dimension. Cronbach’s α was 0.810, and only non-adaptive cognitive emotion regulation was investigated in this study to simplify the model (17).

#### 2.3.4 Acute stress disorder

Stanford Acute Stress Response Questionnaire (SASRQ) was translated by Cardeña, and has 30 items, including dissociative symptoms, avoidance symptoms, repeat symptoms, hypervigilance symptoms, social function impairment in a total of 5 dimensions, with a total score of 0~150 points. The higher the score, the more severe the symptoms of acute disorder (18).

### 2.4 Data collection procedure

Eligible subjects were informed of the purpose of the study, and their personal information would not be disclosed during the study process, and the filling in was completely according to their own wishes. For those with difficulties in filling in the questionnaire, we will objectively read the contents of the questionnaire for some patients and collect data through face-to-face guided communication. After the completion of the questionnaire, the investigator checked whether there were any missing items on the spot and withdrew the questionnaire on the spot. After the questionnaire was completed, the investigator checked whether there were any misses on the spot. If the questionnaire was not completed for various reasons, it was regarded as an invalid questionnaire.

### 2.5 Analysis plan

After sorting and coding, the questionnaires were entered by two people. SPSS 26.0 and AMOS 24.0 software were used for statistical analysis.

(1) Frequency, constituent ratio, mean and standard deviation were used to describe the general information, illness uncertainty, maladaptive cognitive emotion regulation and ASD status.
(2) t test and one-way analysis of variance were used to explore the differences of ASD in the general demographic of the subjects.
(3) Pearson correlation analysis was used to analyze the correlation between illness uncertainty, cognitive emotion regulation and ASD.
(4) Multiple linear regression was used to analyze the influencing factors of ASD.
(5) AMOS 24.0 software was used to construct the structural equation model to explore the interaction path between illness uncertainty, maladaptive cognitive emotion regulation and ASD.

## 3 Results

### 3.1 Sample characteristics

The study included 256 subjects, of whom 145 were male with ischaemic stroke patients between the ages of 26 and 90 (M=64.86 years, SD=11.65). See **Table 1**. The vast majority of patients(94.1%) are treated conservatively. Most of the patients(60.9%) had mild neurological impairment. Most patients’ self-care ability(94.9%) was limited, and only 13 patients did not need to rely on others. See **Table 2**.

**Table 1:** General characteristics of the participants (n=256, %).

Variable	Category	*n* (%)
Gender	Male	145(56.6)
	Female	111(43.4)
Age (years)	M ± SD	64.86 ± 11.65
	< 60	100(39.1)
	≥ 60	156(60.9)
Ethnic groups	Han nationality	152(59.4)
	Ethnic minorities	104(40.6)
Marital status	Married	198(77.3)
	Unmarried	58(22.7)
Education	Primary school and below	72(28.1)
	Junior high school	63(24.6)
	High school/technical secondary school	66(25.8)
	College or above	55(21.5)
Career	Yes	84(32.8)
	No	172(67.2)
Place of residence	Cities	147(57.4)
	Rural areas	109(42.6)
Per capita monthly income	< 2000	32(12.5)
(yuan)	2000-4000	102(39.8)
	≥ 4000	122(47.7)
Medical insurance	Yes	221(86.3)
	No	35(13.7)
Personality	Introversion	125(48.8)
	Extroversion	131(51.2)
Family support	Yes	203(79.3)
	No	53(20.7)

**Table 2:** Disease-related characteristics of the participants (n=256, %).

Variable	Category	*n* (%)
Treatment method	Conservative treatment	241(94.1)
	Thrombolytic/thrombectomy therapy	15(5.9)
Degree of neurological deficit	< 5 points	156(60.9)
(NIHSS score)	≥5 points	100(39.1)
Self-care ability (BI score)	≤40 points	26(10.2)
	41-60 points	54(21.1)
	61-99 points	163(63.6)
	100 points	13(5.1)

### 3.2 Disease uncertainty, cognitive emotional regulation and ASD levels

The mean ASD score among the hospitalized patients with stroke was 53.62 (SD=26.86), which was at a high level. According to Cardena et al. (2000), ASD score of 40 points or higher can predict the occurrence of acute stress disorder in the later stage, with high sensitivity and specificity. In this study, The total score of ASD was 40 or higher in 67.6% of the patients. Specific results for the subdimensions of the SASRQ are shown in **Table 3**. The mean score of disease uncertainty was 72.29(SD=15.59), which was at a medium level. The mean score of non-adaptive cognitive-emotional regulation was 45(SD=12.75). There were 73 patients with moderate ASD, accounting for 28, 5%, and 100 patients with severe ASD, accounting for 39, 1%. So patients with ASD symptoms accounted for 67, 6% in this survey. See **Tables 3**, **4**.

**Table 3:** Scores of ASD, disease uncertainty, and non-adaptive cognitive-emotional regulation.

Variable	Total score	Item mean score
	M ± SD	M ± SD
ASD	53.62 ± 26.86	1.79 ± 0.90
Symptoms of hypervigilance	11.52 ± 6.63	1.92 ± 1.10
Symptoms of avoidance	11.09 ± 5.93	1.85 ± 0.99
Social function impairment	3.49 ± 1.86	1.75 ± 0.93
Symptoms of dissociation	17.34 ± 8.54	1.73 ± 0.85
Relive the symptoms	10.18 ± 4.98	1.70 ± 0.83
Disease uncertainty	72.29 ± 15.59	2.89 ± 0.62
Lack of clarity	39.41 ± 13.39	2.63 ± 0.89
Complexity	25.25 ± 8.18	2.53 ± 0.82
non-adaptive cognitive-emotional regulation	45.00 ± 12.75	2.81 ± 0.80
catastrophizing	10.39 ± 4.61	2.60 ± 1.15
Contemplation	9.80 ± 4.72	2.45 ± 1.18
Self blame	9.55 ± 3.70	2.39 ± 0.92
Blame others	9.43 ± 4.57	2.36 ± 1.14

**Table 4:** The level of ASD of the subjects (n=256, %).

variable	classification	Score range (points)	Number of cases	Ratio of composition
ASD	none	<40	83	32.4
	mild	40~56	73	28.5
	severe	57~150	100	39.1

### 3.3 Univariate analysis of ASD of the participants

The results showed that the ASD scores of stroke patients were different in gender, age, marital status, working status, family monthly income per capita, personality, satisfaction with family support, treatment methods, degree of neurological deficit, and self-care ability, with statistically significant differences (*p*< 0.05). See **Table 5**.

**Table 5:** Univariate analysis of ASD of the participants.

Item	Group	Score	t/F	*P*	LSD
Gender	Male	50.00 ± 22.59	-2.392	0.018	
	Female	58.36 ± 31.06			
Age (years)	< 60	58.12 ± 28.25	2.159	0.032	
	≥ 60	50.74 ± 25.62			
Ethnic groups	Han nationality	54.12 ± 26.52	0.369	0.712	
	Ethnic minorities	52.85 ± 27.51			
Marital status	Married	51.35 ± 25.18	-2.264	0.026	
	Unmarried	61.40 ± 30.94			
Education	Primary school and below	56.46 ± 29.23	0.488	0.691	
	Junior high school	53.08 ± 27.88			
	High school/technical secondary school	50.97 ± 25.70			
	College or above	53.73 ± 24.03			
Career	Yes	60.11 ± 25.71	2.732	0.007	
	No	50.46 ± 26.92			
Place of residence	Cities	53.53 ± 28.07	-0.065	0.948	
	Rural areas	53.75 ± 25.27			
Per capita monthly income	< 2000	84.13 ± 25.88	26.021	<0.001	①>②③
(yuan)	2000-4000	49.11 ± 29.27			
	≥ 4000	49.40 ± 18.79			
Medical insurance	Yes	50.97 ± 25.15	2.356	0.097	
	No	62.29 ± 30.74			
Personality	Introversion	78.47 ± 25.27	9.360	<0.001	①>②③
	Extroversion	51.49 ± 26.79			
Family support	sufficient	49.56 ± 24.24	-4.311	<0.001	
	insufficient	69.19 ± 30.75			
Treatment method	Conservative treatment	52.39 ± 25.92	-2.351	0.033	
	Thrombolytic Procedures	73.53 ± 34.24			
Degree of neurological deficit	< 5 points	49.29 ± 25.29	-3.287	0.001	
(NIHSS score)	≥5 points	60.39 ± 27.95			
	≤40 points	71.73 ± 30.35			
Self-care ability	41-60 points	51.87 ± 25.55	4.737	0.003	①>②③④
(BI score)	61-99 points	46.69 ± 29.10			
	100 points	46.69 ± 29.10			

### 3.4 Correlations between disease uncertainty, cognitive emotion regulation and ASD

Disease uncertainty is positively correlated with non-adaptive cognitive-emotional regulation in stroke acute patients (*r*=0.35, *p*< 0.001). Disease uncertainty is positively correlated with acute stress disorder in stroke patients (*r*=0.47, *p*< 0.001). Acute stress disorder was positively correlated with non-adaptive cognitive-emotional regulation (*r*=0.55, *p*<0.001). See **Table 6**.

**Table 6:** Correlations between disease uncertainty, non-adaptive cognitive-emotional regulation and ASD.

Variable	Disease uncertainty	non-adaptive cognitive emotion regulation	ASD
r (p)			
disease uncertainty	1		
non-adaptive cognitive emotion regulation	0.35 (<.001)	1	
ASD	0.47 (<.001)	0.55 (<.001)	1

### 3.5 Predictors of acute stress disorder

Changes in R^2^ values were observed so as to assess the contribution of general demographic characteristics, disease uncertainty, and cognitive-emotional regulation to the acute stress disorder. The results showed that on top of the original variables, the inclusion of after cognitive emotion regulation (adaptive, non-adaptive), the variance in acute stress disorder increased again by 13.3%, and all variables together explained 56.1% of the total variance in acute stress disorder in the study participants, with the regression equation F=24.295, *p*<0.01, and the results are shown in **Table 7**.

**Table 7:** Predictors of acute stress disorder (N=256).

Variables	Model 1		Model 2		Model 3	
	*β*	*P*	*β*	*P*	*β*	*P*
Gender	0.104	0.052	0.085	0.078	0.050	0.243
Age	-0.134	0.014	-0.124	0.012	-0.112	0.010
Marital status	0.108	0.043	0.098	0.043	0.063	0.137
Work status	-0.180	0.001	-0.160	0.001	-0.122	0.005
Per capita monthly household income	-0.263	<0.001	-0.185	<0.001	-0.156	0.001
Personality	-0.372	<0.001	-0.342	<0.001	-0.300	<0.001
Satisfaction with family support	0.241	<0.001	0.191	<0.001	0.136	0.002
Treatment modality	0.015	0.787	0.032	0.529	0.022	0.626
Degree of neurological deficit	0.175	0.001	0.162	0.001	0.138	0.001
Self-care ability (BI score)	-0.077	0.153	-0.094	0.053	-0.076	0.076
disease uncertainty			0.367	<0.001	0.227	<0.001
Adaptive cognitive emotion regulation					-0.258	<0.001
Non-adaptive cognitive emotion regulation					0.202	<0.001
F(*P*)	10.984(<0.001)		16.904(<0.001)		24.295(<0.001)	
*R* ^2^	0.331		0.455		0.585	
Adjusted *R* ^2^	0.301		0.428		0.561	

### 3.6 The mediating role of non-adaptive cognitive-emotional regulation between disease uncertainty and ASD

**Figure 1:**
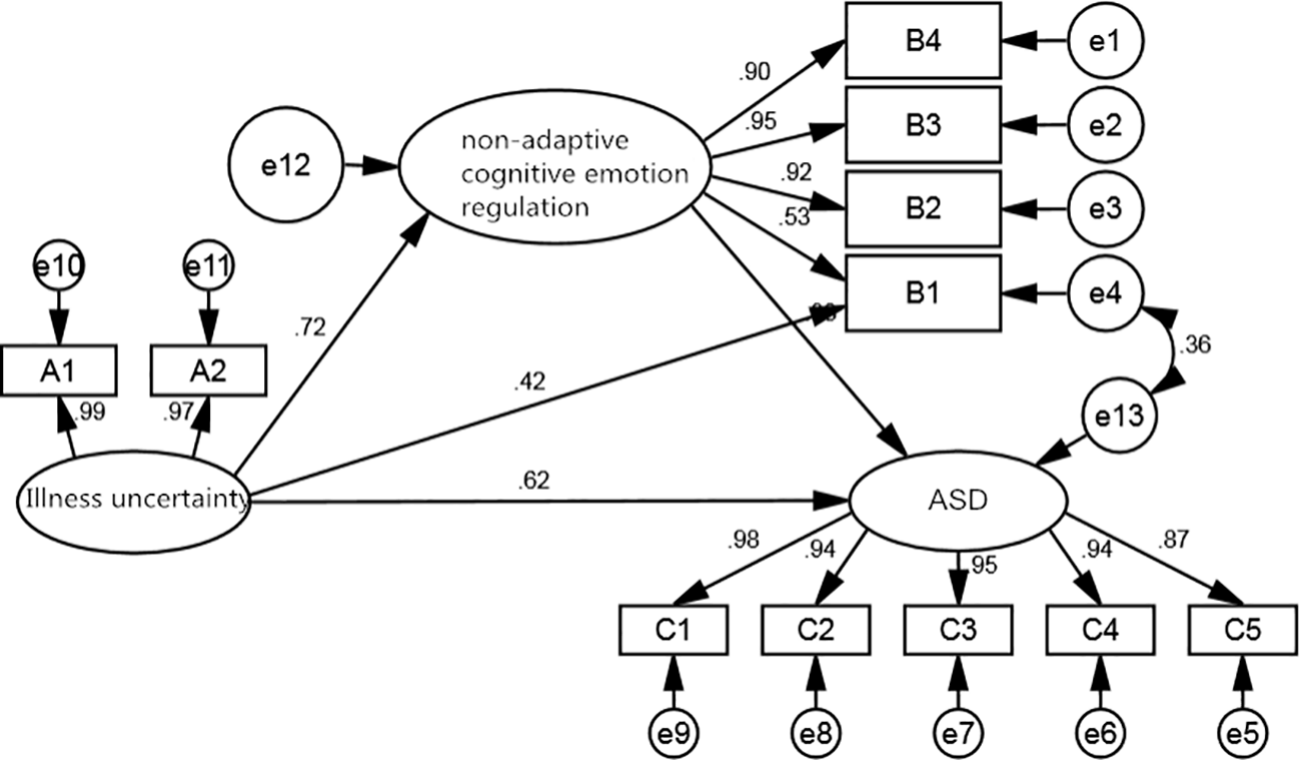
A structural equation model of the mediating role of non-adaptive cognitive emotion regulation.

Considering that non-adaptive cognitive emotion regulation has a positive predictive effect on ASD, this study only explored whether non-adaptive cognitive emotion regulation plays a mediating role. After constructing the mediating effect model, it was found that there were some fitness indicators that did not meet the requirements, so the mediating hypothesis model was revised according to the principle of gradual model revision. Therefore, after the addition of the two correlation paths correction, the structural equation model fit better, and the final model is shown in **Figure 1**, and the model fitting parameters are shown in **Table 8**.

**Table 8:** Structural equation model fitness index values.

indicators	CMIN/DF	GFI	IFI	NFI	AGFI	CFI	RMSEA
Criteria of adaptation	<3.0	>0.9	>0.9	>0.9	>0.9	>0.9	<0.08
Index of fit	2.346	0.939	0.988	0.979	0.897	0.988	0.073

GFI, Goodness of Fit Index; IFI, Incremental Fit Index; NFI, Normed Fit Index; AGFI, Adjusted Goodness of Fit Index; CFI, Comparative Fit Index; RMSEA, Root Mean Square Error of Approximation.

The results of the intermediate effects test showed that the significance tests for all three pathway coefficients reached the 0.05 level of significance. Disease uncertainty was a positive predictor of non-adaptive cognitive emotion regulation (β=0.716, *p*<0.001), non-adaptive cognitive emotion regulation was a positive predictor of ASD (β=0.378, *p*<0.001) and disease uncertainty was a positive predictor of ASD (β=0.617, *p*<0.001). There was both a direct and an indirect effect of illness uncertainty on ASD, where the direct effect was 0.617, the indirect effect was 0.271, and the total effect was 0.888. A Bootstrap was applied to test the hypothesis of mediating effects, and the results showed that the 95% confidence interval for each pathway did not contain 0, further validating the existence of a partial mediation effect. The coefficients and effect values for each path are detailed in **Tables 9** and **10**.

**Table 9:** Coefficients for each path of the structural equation model.

Pathways	Standardized Estimate	S.E.	*P*
Non-adaptive cognitive-emotional regulation <–Disease uncertainty	0.716	0.027	<0.001
ASD <– Non-adaptive cognitive-emotional regulation	0.378	0.017	<0.001
ASD <– Disease uncertainty	0.617	0.009	<0.001
A2 <– Disease uncertainty	0.972		
A1<—Disease uncertainty	0.989	0.034	<0.001
B4<–Non-adaptive cognitive-emotional regulation	0.898		
B3<–Non-adaptive cognitive-emotional regulation	0.945	0.042	<0.001
B2<–Non-adaptive cognitive-emotional regulation	0.922	0.045	<0.001
B1<–Non-adaptive cognitive-emotional regulation	0.526	0.045	<0.001
C5<–ASD	0.869		
C4<—ASD	0.944	0.123	<0.001
C3<—ASD	0.954	0.161	<0.001
C2<—ASD	0.940	0.147	<0.001
C1<—ASD	0.981	0.198	<0.001

**Table 10:** Results of a test of mediating effects of non-adaptive cognitive emotion regulation.

	Estimate	StandardizedEstimate	SE	Z	95%CI				*P*
					Bias Corrected		Percentile		
					Lower	Upper	Lower	Upper	
Indirect effects	0.055	0.271	0.007	7.857	0.043	0.070	0.043	0.069	<0.001
Direct effect	0.126	0.617	0.010	12.600	0.107	0.147	0.106	0.146	<0.001
Total effect	0.181	0.888	0.009	20.111	0.162	0.200	0.162	0.199	<0.001

## 4 Discussion

### 4.1 Sample characteristics

Among 256 patients, 56.6% were male patients, more males than females, in line with the gender characteristics of stroke in China (19). The proportion of male stroke prevalence in this study was slightly higher than the domestic average (20), and the reason for the high proportion of male patients may be related to the higher prevalence of hypertension and hyperlipidaemia and the number of smokers among males in the survey area (21). According to the survey, the incidence of hypertension was 1.3 times higher in men than in women, and the incidence of hyperlipidaemia was three times higher in men than in women, and the incidence of smoking in daily life was also higher in men than in women. The mean age of onset of patients in this study was 64.71(SD=12.20), which is generally consistent with the peak age of onset in the 2019 Chinese Stroke Epidemiological Survey (mean age of onset was 65), and there is a trend towards a younger age of stroke onset in China compared with 2005 (22, 23). In the present study, 39.1% of patients were under 60 years of age, further validating this trend. In terms of marital status, although the majority of patients had spouses, 22.7% still had no spouse. As the affected population is mainly middle-aged and elderly patients, those without spouses need more attention and care in their lives and emotions, therefore, health care workers should pay attention to them and strengthen their life assistance and emotional support.

Only 5.9% of patients opted for thrombolysis or thrombolysis, and most patients still adopted conservative treatment. This may be due to the fact that, on the one hand, the clinical control of patients’ thrombolytic conditions is more strict, and thrombolysis can only be performed after meeting the thrombolytic conditions and treatment time window (24), and on the other hand, patients are worried about taking the risk of thrombolysis and have certain concerns. On the NIHSS score, 39.1% of patients had moderate to severe neurological deficits, and on the self-care BI score, only 5.1% of patients did not need to be dependent, and 10.2% were at the level of severe dependence, indicating that stroke can lead to varying degrees of physical problems and is an important cause of disability in patients (25).

### 4.2 Disease uncertainty, cognitive emotional regulation and ASD levels

In the general context of novel coronavirus pneumonia, most hospitalized patients can experience acute stress reactions of varying degrees after illness due to original role-behavior conflicts, long disease treatment cycles, and economic stress (26). After admission disease patients are prone to sleep disturbances, emotional, memory and other brain neurological disorders due to stress factors, causing a reduction in their own physiological and psychological defenses, further aggravating the acute Stress (27). The diagnostic value of ASD is optimal when the total score reaches the level of 40. In this study, the mean ASD score of stroke patients was 53.62(SD=26.86), and 67.6% had moderate to high levels of acute stress symptoms. Therefore, early identification, attention and intervention by health care professionals are urgently needed, and the follow-up of patients after discharge from community hospitals should be strengthened if necessary to implement linkage Management.

Psychological condition is one of the important factors that cause ASD in patients, among which individuals tend to develop a sense of disease uncertainty due to lack of proper judgment of disease-related knowledge. Stroke patients are prone to a strong sense of disease uncertainty because of their first onset, rapid onset, lack of systematic understanding of the disease, and inability to fully recognize knowledge about the disease (28). Non-adaptive cognitive regulation of stroke patients was at a moderate level. Non-adaptive cognitive regulation can lead to psychological problems such as anxiety and depression, and can exacerbate acute stress levels (29). If patients do not adjust their psychological state in a timely manner, this may lead to choices such as catastrophizing and contemplation, which may affect mental health outcomes.

### 4.3 Predictors of acute stress disorder

#### 4.3.1 Statistically significant variables

The results of multivariate analysis showed that age was an independent risk factor for ASD in stroke patients. The younger the ischaemic stroke patients were, the higher their acute stress levels were. It suggested that younger patients are more prone to psychological stress compared to older patients, which is in line with the findings of another scholar (15). Many studies have found that acute stress symptoms are inversely proportional to age in adult patients. For example, Andrynowski (30) found that ASD symptoms are mild in elderly cancer patients, but more severe in young patients. The elderly are better prepared for the loss of various life, work and social functions after illness, and tend to be more prepared for the possible death caused by illness. They can deeply understand the acute stress, and have relatively strong psychological tolerance, and are relatively stable in material and mental aspects. However, middle-aged and young people experienced less events, had weaker psychological tolerance, were more confused, needed a longer time to accept stressful events, needed a certain time to buffer stressful events, lacked psychological preparation, and worried about the prognosis aggravated their psychological stress.

In our study results, we found that the level of ASD in female stroke patients was significantly higher than that in male stroke patients, which was consistent with the more recognized research results at home and abroad that the incidence of ASD in females was higher than that in males, indicating that female stroke patients were more likely to have a higher level of ASD than male stroke patients. The main reason may be that female patients have a higher susceptibility to mood disorders, and for female patients, the psychosocial stress associated with illness is indeed significantly different from that of men (30). Men bear heavy pressure in the society and pay more attention to work and career, while women play a central role in the family. When facing stress, men have a higher threshold for stress, and women have a lower threshold. After the disease, female patients worry about their own recovery and prognosis, worry about the life of the family and their future work, and often have wild thoughts. Prone to anxiety and fear. It is suggested that we should pay more attention to the female group when providing psychological nursing services for stroke patients.

Furthermore, economic conditions influence patients’ positive affective experiences when seeking medical care and are an important guarantee for patients to treat their illness. Patients with high per capita monthly household income generally have better social support and literacy, better awareness of the disease and access to medical resources, so they have greater confidence in their ability to cope with the disease, have better psychosocial adjustment and have relatively low levels of acute stress; conversely, most of low-income families were worried about not being able to afford treatment for the disease, were under great psychological stress, resulting in higher levels of acute stress. It is suggested that health care workers focus on the economically disadvantaged, provide the right emotional support, provide timely comfort and emotional relief, and encourage patients to release their stress in order to reduce acute stress levels.

As you can see from the table, personality can have a significant impact on acute stress levels in ischaemic stroke patients, with introverted patients having significantly higher levels of acute stress than extroverted patients. Personality is a stable core personality trait that individuals display in the face of the external environment, and has a great influence on their level of mental health. It is influenced by a combination of congenital genetics, acquired experiences and life environment, and does not change significantly over time, so individuals can only make limited adjustments to it within a certain range. Patients with extroverted personalities are more optimistic, positive and optimistic, with stronger communication and adaptation skills, and focus more on obtaining information that is beneficial to their recovery after illness. This can lead to increased levels of acute stress. Therefore, health care professionals need to focus on the emotional state of introverted patients and develop individualized psychological intervention strategies according to the actual situation, so that patients can change their negative attitude as soon as possible and reduce the level of acute stress.

Apart from that, Patients with stroke experience varying degrees of neurological deficits, and the recovery period for stroke neurological deficits is relatively long and prone to sequelae, especially in patients with severe neurological deficits, where mobility is severely restricted, powerlessness is particularly evident, highly stressful emotional reactions are present and acute stress levels are higher. This suggests that health care professionals should encourage and guide patients to undertake rehabilitation exercises as early as their condition allows, to promote early recovery of neurological function, improve their prognosis and prevent an increase in acute stress levels.

The higher the level of uncertainty in illness and the more inclined to adopt non-adaptive cognitive emotion regulation, the more severe the acute stress response. The complexity of stroke treatment and the longer treatment period can stimulate patients to develop disease uncertainty. High levels of disease uncertainty can lead to increased symptoms such as anxiety, irritability, and hypervigilance, which increase the level of acute stress response and thus affect prognosis and quality of life (31). In addition, psychological stress theory suggests that cognitive factors play an important role in the stress system and that the degree of acute stress response in a stressful situation depends on the cognitive evaluation and subsequent emotional regulation of the individual in response to the stressful event (32). Different approaches to cognitive emotion regulation will influence the level of stress in individuals. Acute stress symptoms such as fear, anxiety, and hypervigilance are more severe in patients who frequently use non-adaptive cognitive-emotional regulation (33).

#### 4.3.2 Variables that are not statistically significant

In this study, ethnicity, education level, place of residence, and whether they have medical insurance have no statistical significance in stroke ASD, which are not predictive variables for ASD in stroke patients in this study. The results may be different from the studies of other scholars, and the reason may be related to the differences in sample size and study area. In this study, the sample size of the survey is relatively small, and the ethnic representation of the survey area may be relatively weak, so the influence of ethnicity on ASD is not significant.

In our study, educational level as a predictor of ASD has not been confirmed, which may be affected by the sample size and sample characteristics bias. However, some studies have shown that the higher the education level, the less likely to develop ASD (34). We believe that patients with high education level have better learning ability, can quickly understand the relevant knowledge of the disease, make full use of their own social resources and resources around them, and in the face of stressful events, they can better control their negative emotions with calm thinking, find a reasonable way to vent, and have better psychological adjustment ability, so they show milder ASD symptoms.

Some studies suggest that patients living in rural areas are more likely to develop ASD, people living in urban areas have better medical resources in tertiary hospitals, rural medical environment lags behind urban areas, and patients lack relevant knowledge. This study shows that residence is not related to the occurrence of ASD in stroke patients, which may be related to the severity of symptoms in the affected population. Many rural patients do not receive treatment in rural or county hospitals with backward medical environment, and choose to receive treatment in tertiary hospitals, which improves the confidence of patients in treatment. Economic status was relatively better, so there was no difference in the occurrence of ASD by residence.

The economic burden of patients with medical insurance protection is small, while most of the medical expenses of patients are paid by themselves. The economic and psychological burden of patients and their families is heavy, and the disease limits their behavioral ability, unable to provide material guarantee for their families and themselves, and easy to produce mental illness. However, we did not find that the payment method of medical expenses was the influencing factor of ASD in our study, which may be related to the different policies of the surveyed countries, regions, or the small sample size.

### 4.4 The mediating role of non-adaptive cognitive-emotional regulation

The mediating effect test verified that non-adaptive cognitive-emotional regulation in stroke patients played a significant role in the pathway of the effect of disease uncertainty on ASD, with a mediating effect of 30.5%, suggesting that disease uncertainty can exacerbate the level of acute stress through the patient’s choice of non-adaptive regulation. Illness uncertainty can be an important stressor influencing the stress response and directly affects the patient’s ability to adapt to stress in a stressful environment. When the level of disease uncertainty reaches a certain level under the stimulus of a stressor, it can stimulate the body to inspire stress defense mechanisms, which can exacerbate the stress and lead to poor outcomes (35). Patients with stroke are sensitive to the perception of illness and emotions when faced with a sudden illness, and are prone to brooding, anxiety, avoidance, and negativity. They tend to adopt avoidance and negative attitudes. If negative emotions cannot be expressed through a reasonable way, patients cannot correctly open their own self-regulation mode, which is easy to lead to poor coping and the choice of non-adaptive regulation. Thus, psychological defense ability is weakened and acute stress levels are increased. Buehler (36) proposed and verified two kinds of coping styles and their effects in dealing with negative emotions by experimental methods: ruminative, immersed in the negative aspects of the passive self, forming a susceptible type of depression; reflective– exploratory, active, open to the self, can restore to the state of mental health as soon as possible. In the face of stressful events, individuals with attentive coping style are prone to produce negative emotions and thus produce more negative evaluations of themselves, while individuals with reactive coping style can produce more positive thoughts about themselves (29). The characteristics of indulging in fantasy, letting go of thoughts and emotions, and pursuing strong emotions make people more inclined to repeatedly review the situation and feelings at the time, and think things too bad, which leads to the appearance or even aggravation of ASD symptoms. There is no significant direct effect of illness uncertainty on ASD, but it has a significant predictive effect on ASD by reducing the use of negative cognitive emotion regulation strategies and promoting the use of positive cognitive emotion regulation strategies. Because patients with strong illness uncertainty are sensitive and passive in personality, they believe that they cannot change the status quo after illness, repeatedly review the experience at that time, and repeatedly think about how bad things are, and tend to adopt more negative coping strategies. They are unable to look at problems from a new perspective, which leads to the aggravation of ASD symptoms. Therefore, medical staff should focus on patients’ mental state (37), actively guide patients to express their uncertainty in illness, and timely identify the danger signals of non-adaptive cognitive emotions. As soon as possible, cognitive intervention therapy, cognitive course learning, health education and psychological nursing should be adopted to correct patients’ non-adaptive cognitive regulation and change their negative cognitive emotions. Cultivate adaptive cognitive behavior, so as to achieve the goal of reducing the level of acute stress.

## 5 Conclusion

This study investigated the mediating role of non-adaptive cognitive-emotional regulation. We found that disease uncertainty and cognitive emotion regulation are important in influencing the acute stress response of stroke patients. Therefore, in the global epidemic virus outbreak situation, healthcare professionals should focus on the psychological state of patients, actively guide patients to express disease uncertainty, and at the same time, identify the danger signals of non-adaptive cognitive emotions in a timely manner, and take cognitive interventions as early as possible to transform poor cognitive emotions, reduce the level of acute stress and promote disease recovery.

## 6 Limitations

Our study included a majority of participants older than 60 years of age, which may have limited representative ability. We have excluded patients with mental disorders, so they cannot be used for this type of population. Another limitation is the nationality of the investigated population, which has certain geographical and ethnic limitations because our study population is only Chinese people.

## 7 Implications for practice

Study findings provide important information to guide future practice for nurses and other health-care professionals. For the future, routine screening of acute stress symptoms in stroke patients is extremely necessary, and mental health support should be delivered in a timely fashion for stroke patients. Clinical medical staff should pay attention to the regulation of the mental state of stroke patients, construct individualized stress intervention measures, scientifically guide ischemic stroke patients to reduce the disease uncertainty, correct the wrong cognition of patients, and cultivate adaptive cognitive behavior, so as to achieve the goal of reducing the level of ASD.

## Data availability statement

The original contributions presented in the study are included in the article/supplementary material. Further inquiries can be directed to the corresponding author.

## Ethics statement

The studies involving humans were approved by Medical Ethics Committee of Yanbian University School of Medicine. The studies were conducted in accordance with the local legislation and institutional requirements. The participants provided their written informed consent to participate in this study.

## Author contributions

YaZ: Conceptualization, Data curation, Formal analysis, Investigation, Methodology, Project administration, Supervision, Writing – original draft. YiZ: Conceptualization, Data curation, Investigation, Project administration, Writing – original draft.
